# Electronic structure engineering of PVA/ZnO/graphene oxide nanocomposites: a DFT study toward CO₂ and humidity detection

**DOI:** 10.1038/s41598-026-62966-6

**Published:** 2026-07-24

**Authors:** Asmaa Ibrahim, Mervat Abd El Aal, Hayam El-Zahed, Ahmed F. Mabied

**Affiliations:** 1 Physics Department, Faculty of Women for Arts, Science and Education, Ain Shams University, Cairo, 11757 Egypt; 2 Physics Department, Faculty of Science, Galala University, New Galala City, Suez, 43511 Egypt; 3 X-ray Crystallography lab, Solid State Physics Department, National Research Center, 33 Bohouth St, Dokki, Giza, 12622 Egypt

**Keywords:** Polymer Nanocomposite, DFT, Energy gap, QTAIM and NCI analysis, MESP and Global reactivity, Chemistry, Materials science, Nanoscience and technology

## Abstract

Due to their adjustable physicochemical properties and easy incorporation with functional nanomaterials, nanocomposites based on polyvinyl alcohol (PVA) have garnered significant interest for gas and humidity sensing applications. This study systematically examined the structural, electronic, adsorption, and sensing-related properties of PVA/ZnO/graphene oxide (GO) nanocomposites using density functional theory (DFT) at the B3LYP/LanL2DZ level. Strong interfacial interactions and hydrogen-bond-assisted stabilization within the nanocomposite structure were revealed by the calculated infrared spectra, molecular electrostatic potential (MESP), quantum theory of atoms in molecules (QTAIM), and non-covalent interaction (NCI) analyses. The electronic properties of PVA were significantly modified by the addition of ZnO and GO, as demonstrated by a reduction in the HOMO -LUMO energy gap from 7.334 eV to 1.075 eV and an increase in the total dipole moment from 7.147 to 12.243 Debye, which suggests that charge transfer and electronic polarization have been enhanced. Adsorption studies on H₂O and CO₂ molecules revealed that interactions are thermodynamically favorable, with adsorption energies of -0.306 eV and − 0.381 eV, respectively. PVA/OZn/GO–CO₂ showed the smallest energy gap (0.539 eV) and the largest dipole moment (14.264 Debye) among all configurations examined, indicating a marked electronic responsiveness and potential applicability in gas sensing. The analysis of the density of states further substantiated the emergence of electronic states that promote charge transport and enhance conductivity upon adsorption. The incorporation of ZnO/GO is offers an effective strategy for designing potential PVA-based nanocomposites for CO₂ gas and humidity sensing applications, as evidenced by the combined electronic modulation, strong adsorption affinity, and favorable charge redistribution.

## Introduction

The rapid progress in materials science and nanotechnology has enabled the development of advanced functional materials with tunable structural and electronic characteristics. Conventional gas and humidity detection systems, however, often suffer from limitations such as low selectivity, slow response, and sensitivity to environmental fluctuations. Incorporating nanomaterials into polymer matrices offers an effective strategy to overcome these challenges by enhancing properties such as surface area, electronic conductivity, and interfacial activity. Polyvinyl alcohol (PVA), in particular, is valued for its versatility, environmental compatibility, and ability to incorporate diverse functional nanomaterials, making it a strong foundation for designing multifunctional nanocomposites with improved electronic and interfacial performance^1–3^.

In order to attain improved functional qualities, polymeric nanocomposites are hybrid materials made up of nanoscale fillers scattered throughout polymer matrix. These materials combine the ease of manufacture, flexibility, and affordability of polymers with the complex electrical and optical properties of nanoparticles. Sensitivity can be raised by incorporating metal oxides like ZnO and carbon-based substances like graphene oxide (GO) into sensor technology^4–6^.

The water-soluble synthetic polymer PVA is well known for its mechanical stability, film-forming capabilities, and biodegradability. Strong hydrogen bonds are made possible by its high hydroxyl group density, which also makes it compatible with a wide range of nanofillers. The manufacturing and longevity of sensor devices depend on the desirable dielectric, flexible, and chemically stable characteristics of PVA-based films^7–10^.

ZnO is a widely studied n-type semiconductor characterized by a direct wide bandgap (3.37 eV) and high exciton binding energy (60 meV). It also features notable optical and piezoelectric properties^11,12^. The high surface-area-to-volume ratio of ZnO nanoparticles enhances their gas adsorption capabilities, making them effective in detecting hydrogen, ammonia, and volatile organic compounds^13,14^.

GO is a chemically modified derivative of graphene that preserves its outstanding surface area and conductivity while offering abundant oxygen-containing functional groups. These groups facilitate uniform dispersion within polymer matrices and improve mechanical properties, chemical sensitivity, and analyte adsorption. In nanocomposites, GO promotes tunneling and hopping conduction mechanisms that enhance sensor responsiveness^15–18^.

Combining ZnO and GO in a PVA matrix results in a synergistic multifunctional nanocomposite. The interactions between them lead to improved gas selectivity, faster response and recovery times, and heightened durability key attributes for advanced sensing systems whereas GO introduces additional conductive networks and mechanical reinforcement^19–21^.

Gas sensors operate through chemiresistive mechanisms, where gas interactions with the sensor surface cause changes in resistance. For humidity sensing, hydrophilic materials like PVA change their dielectric constant and ionic conductivity due to water vapor adsorption, while metal oxides and GO enhance performance by increasing binding sites and optimizing charge transport^22–24^.

Molecular modeling techniques such as Density Functional Theory (DFT) offer insights into molecular interactions between PVA and nanofillers. These methods allow prediction of structural and electronic properties, including charge transfer, energy band alignment, and composite stability. Modeling can also forecast how ZnO interact with GO and PVA at the atomic level, guiding experimental design and explaining observed sensing behaviors^25–27^. Density Functional Theory (DFT) and its time-dependent variant, TD-DFT, have been used quite a lot to probe the structural, electronic, optical, and adsorption characteristics of nanomaterials, offering really helpful glimpses into the way structure links to properties and how charge-transfer processes happen^28,29^.

Although numerous studies have explored the individual reportedof ZnO, or GO into PVA, the combined incorporation of the two nanomaterials into a single matrix for dual sensing applications remains underexplored. Furthermore, there is a lack of comprehensive correlation between computational modeling and experimental results in existing literature.

Therefore, the novelty of this work lies in the development of a PVA/ZnO/GO hybrid nanocomposite for dual sensing applications. This study provides a comprehensive theoretical insight into the structural, electronic, and adsorption properties of PVA/ZnO/GO nanocomposite using Density Functional Theory (DFT) using the function B3LYP/LanL2DZ calculations. A direct correlation was established between the nanocomposite electronic structure modulation (energy gap and dipole moment variations, MESP, QTAIM, and NCI analyses) and its adsorption performance towards the target analytes, H₂O and CO₂, to provide a detailed understanding of the sensing mechanism. These results lay the theoretical groundwork for the rational design of PVA-based nanocomposite with tailored properties for gas and humidity sensing applications. Overall, the findings provide a solid molecular-level foundation to guide the development of PVA-based nanocomposite for advanced sensor technologies, with emphasis on conceptual design rather than practical cost or environmental claims.

## Computations details

All quantum chemical calculations for the PVA//ZnO/GO structures were conducted utilizing the Gaussian 09 software package^30^, applying the B3LYP functional alongside the LanL2DZ basis set^31–33^. B3LYP/LanL2DZ level of theory was employed due to its reliability in describing the structural and electronic properties of nanostructured systems^34^.Without imposing symmetry requirements, geometry optimizations were carried out, and vibrational frequency analysis was used to confirm that the optimized structures were indeed minima. The total dipole moment (TDM), energy gap (ΔE = HOMO-LUMO), molecular electrostatic potential (MESP), infrared (IR) spectra, and both the density of states (DOS) and projected density of states (PDOS) were evaluated, among other electronic and structural characteristics. Additionally, to obtain a more profound understanding of the interactions within the composite systems, Quantum Theory of Atoms in Molecules (QTAIM) analysis was conducted with both Multiwfn and VMD software^35,36^ to test the stability of the studied model structures. Non-covalent interactions (NCI) were also investigated through reduced density gradient (RDG) analysis to illustrate weak intermolecular forces such as hydrogen bonding and van der Waals interactions. Moreover, global reactivity descriptors such as ionization potential (I), electron affinity (A), chemical potential (µ), hardness (η), softness (S), and electrophilicity index (ω) were calculated to assess the overall chemical reactivity and stability of the studied complexes, as defined by the following equations^37^:

I= -E_HOMO_.

A= - E_LUMO_.

µ= -(I + A)/2.

η=(I-A)/2.

S = 1/η.

ω = µ^2^/2η.

## Results and discussion

### Billing model

**Fig. 1:**
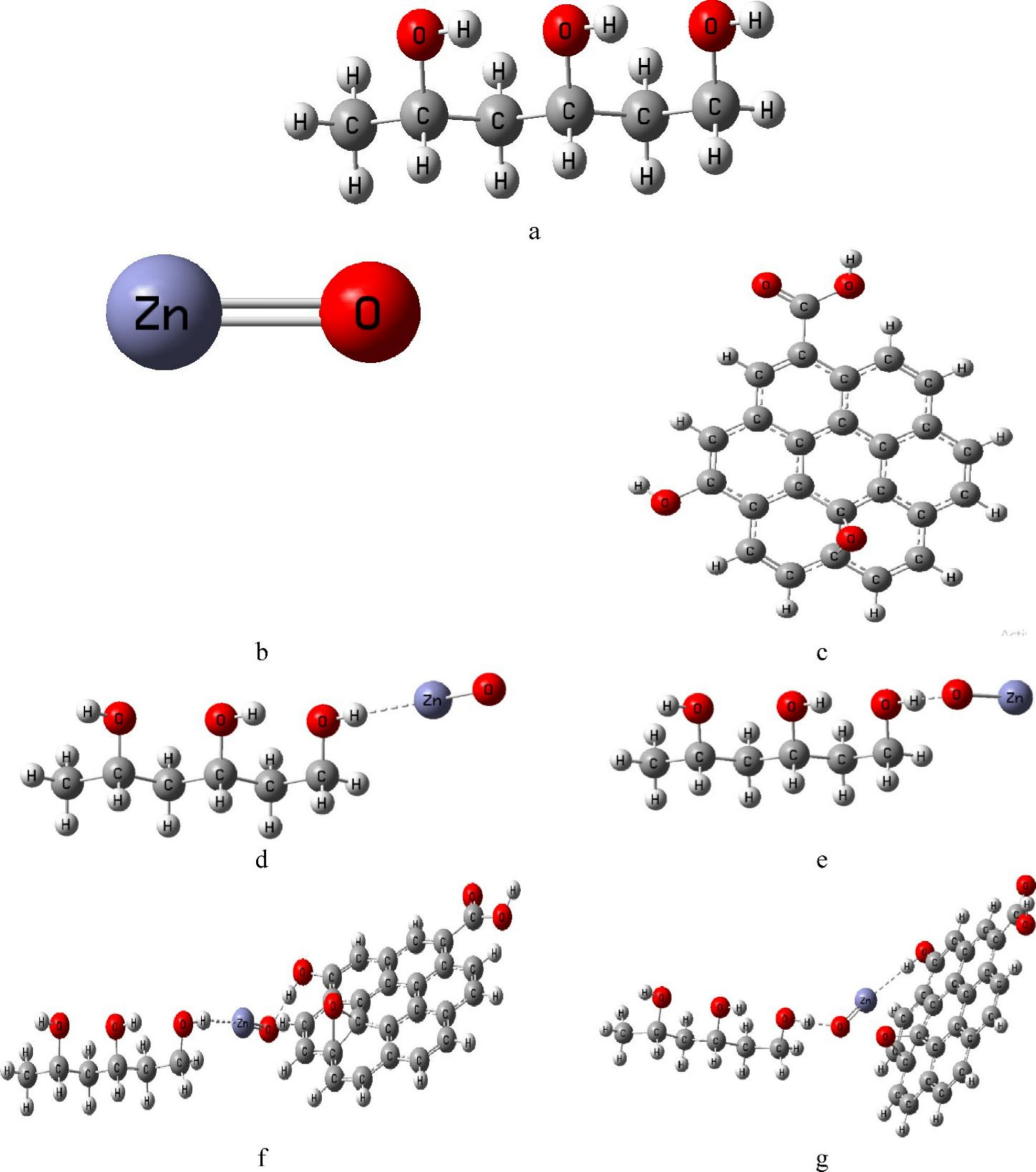
Module molecules structures for (**a**) PVA, (**b**) ZnO, (**c**) Graphene oxide (GO), (**d**) PVA/ZnO, (**e**) PVA/OZn, (**f**) PVA/ZnO/GO and (**g**) PVA/OZn/GO.

DFT previous studies have demonstrated that three repeating units (trimers) reliably reproduce the electronic behavior of extended polymer chains while offering significant computational efficiency^38^. The molecular model was constructed utilizing trimers of polyvinyl alcohol (PVA), as depicted in Fig. 1-a. Zinc oxide (ZnO), illustrated in Fig. 1-b, exhibits weak interactions with the hydroxyl (OH) groups of PVA through both the zinc and oxygen atoms of ZnO, as demonstrated in Figs. 1-d and -e, respectively. Graphene oxide (GO), represented in Fig. 1-c, was integrated under the assumption of weak interactions between its OH groups and the PVA/ZnO complex. These interactions take place through both the O and Zn atoms of ZnO, as shown in Figs. 1-f and -g, respectively.

**Fig. 2:**
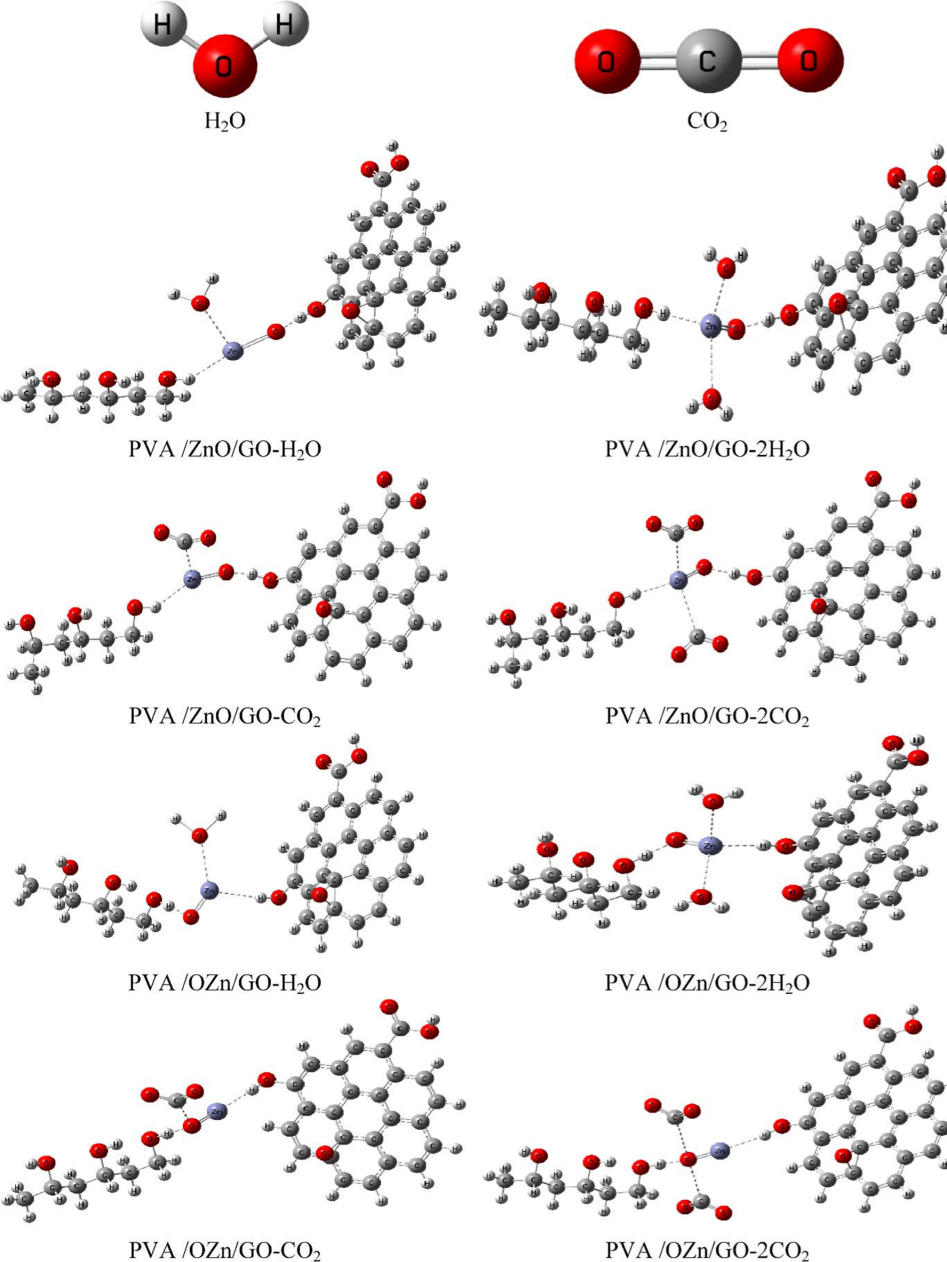
Module molecules structures for the interaction of PVA/ZnO/GO and PVA/OZn/GO with H_2_O and CO_2_as well as 2H_2_O and 2CO_2_.

Figure 2 displays the optimized molecular structures that illustrate the weak interaction of the PVA/ZnO/GO composite with one and two molecules of water (H₂O) and carbon dioxide (CO₂). The weak interaction was modeled from two perspectives: once through the oxygen atom of ZnO and once through the Zn atom for both the PVA/ZnO/GO and PVA/OZn/GO structures. This methodology aims to investigate the sensitivity of the composites to both humidity and gas exposure. All the model molecules were built with Gaussian 5.0 software^39^.

## Calculated IR

**Fig. 3:**
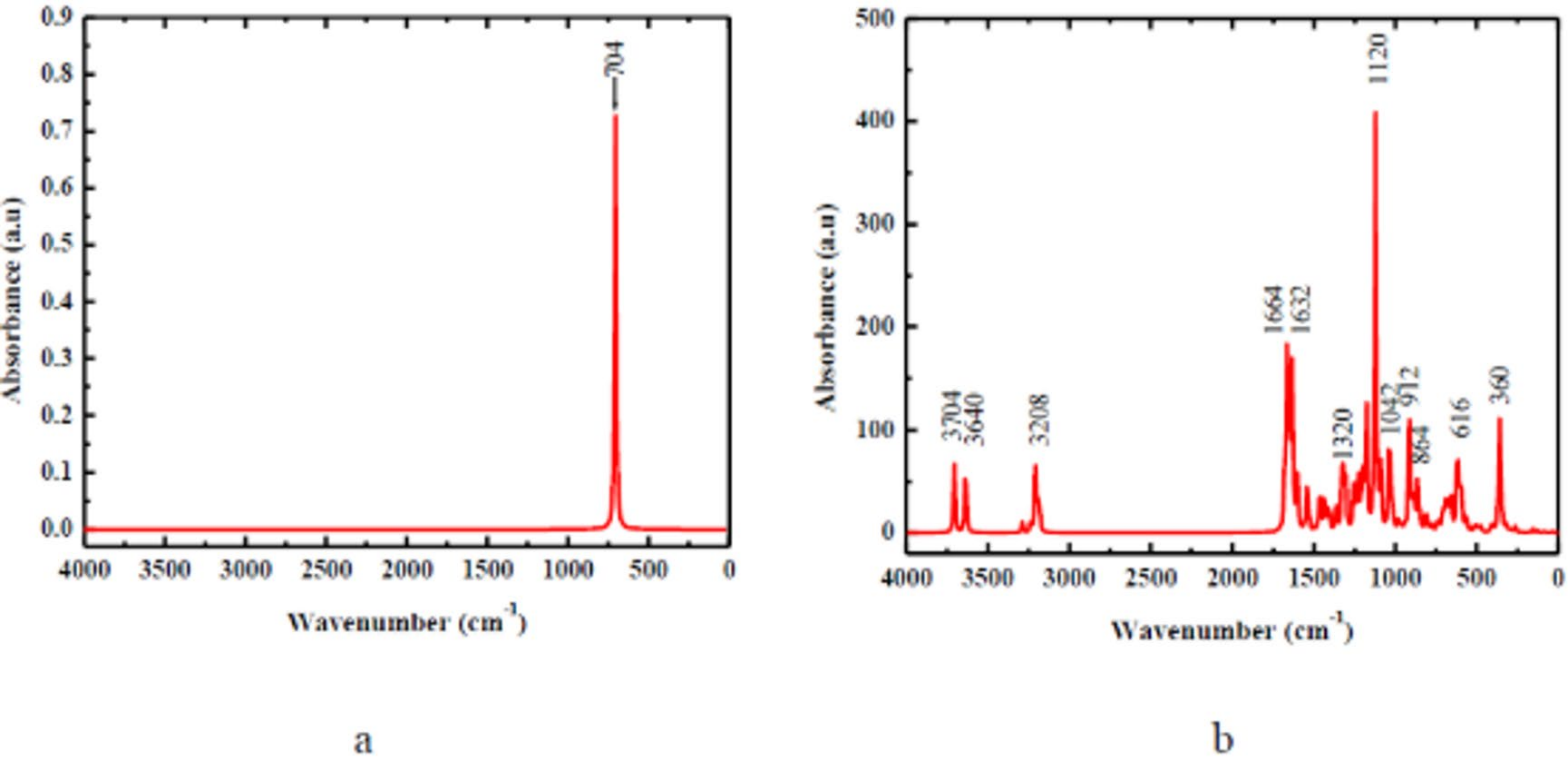
B3LYP/LanL2DZ calculated IR absorbance spectra for a-ZnO and b- GO.

Figure 3 illustrates the calculated IR spectra of ZnO and GO, with theoretical calculations conducted using the B3LYP/LanL2DZ method. Figure 3-a presents a strong absorption peak at 704 cm^−^¹ due to Zn–O stretching. The GO spectrum (Fig. 3-b) exhibits absorption bands at 3704, 3640, 3208, 1664, 1632, 1320,1120, 1042, 912, 864, 616, and 360 cm^−^¹. These band are attributed to the stretching vibrations of O–H (3704–3640 cm^−^¹), O-H hydrogen bond stretching (3208 cm^−^¹), C = O (1664 cm^−^¹), C = C (1632 cm^−^¹), and C–O bonds (360–1320 cm^−^¹), indicating the presence of various oxygen-containing functional groups on the GO surface. The calculated spectra for both ZnO and GO demonstrates the reliability of the DFT-based B3LYP/LanL2DZ method in predicting vibrational features and provides strong evidence for the structural models employed^40,41^.

**Fig. 4:**
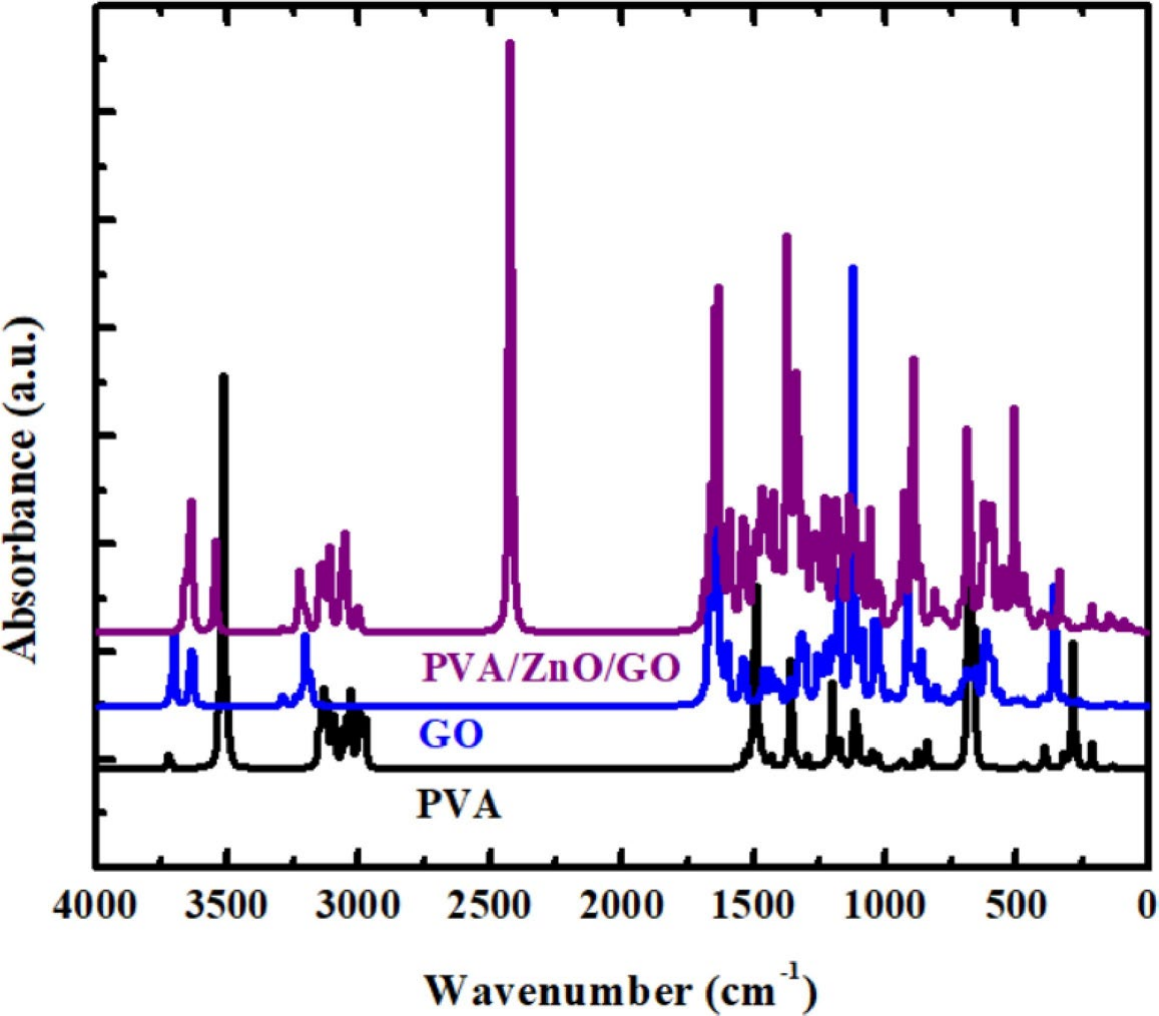
B3LYP/LanL2DZ calculated absorbance spectra for PVA, ZnO, GO and PVA/ZnO/GO.

Figures 4 illustrate the calculated IR spectra of PVA along with its composite mixtures containing ZnO NPs and GO using the B3LYP/LanL2DZ method. The spectra reveal characteristic absorption bands for PVA in the range of 3300–3500 cm⁻¹, which are attributed to O-H stretching vibrations, indicating strong hydrogen bonding within the polymer matrix. Additional band observed at approximately 2900 cm⁻¹ are linked to C-H stretching vibrations, while sharp band found between 1000 and 1300 cm⁻¹ correspond to C-O stretching and C-H bending modes. Significant changes are brought about by the addition of ZnO and GO, including increased intensity and minor shifts in the O-H and C-O stretching bands, indicating strong interactions between the nanofillers and the polymer matrix. Notable bands associated with O-H, C-H, and C-O stretching modes are observed, with slight variations in wavenumber attributed to computational approximations.

Overall, the staying power of the characteristic vibration bands and the lack of any negative frequencies, gives a good sign that the optimized PVA/ZnO/GO structures are energetically steady and they really land in true minima, not just in something like transition states.

## TDM and energy gap

The TDM values indicate the intensity of electronic transitions, with higher values signifying more effective electron excitation. The energy gap HOMO-LUMO (ΔE) denotes the energy difference between the highest occupied molecular orbital and the lowest unoccupied molecular orbital, which affects the electronic and optical characteristics of the material. Table 1 displays the computed values of the TDM and the energy gap for the analyzed structures. The energy gap and TDM are key descriptors that directly relate to the electrical response of sensing materials. A reduced energy gap lowers the energy barrier for charge transfer between the sensing layer and adsorbed gas molecules, facilitating carrier modulation and resulting in measurable changes in electrical resistance. Similarly, an increased TDM indicates enhanced electronic polarizability and stronger interfacial charge redistribution upon gas adsorption, which affects the dielectric properties of the sensing layer and leads to observable capacitance or impedance variations. Thus, the combined decrease in the HOMO–LUMO gap and increase in TDM provide a quantitative mechanistic link between molecular-level interactions and macroscopic sensor outputs.

As illustrated in the table, the values show considerable variation among the samples and a reduction in the energy gap with the incorporation of ZnO and GO into the PVA is observed. The reduction in the energy gap with ZnO/GO coming in suggests better electronic delocalization and a kind of charge transfer ability, is help for adsorption, as in modulating the electronic behavior^42^. Significantly, the PVA/OZn/GO composite demonstrates the lowest energy gap of 1.075 eV, suggesting a greater ease of electronic excitation, along with a relatively high TDM value of 12.243 Debye. This combination of a high TDM and a low energy gap implies that PVA/OZn/GO possesses superior electronic properties in comparison to the other structures examined. This enhanced reactivity renders the PVA/OZn/GO composite particularly well-suited for use as a gas or humidity sensor.

**Table 1:** B3LYP/LanL2DZ calculated TDM as Debye and HOMO/LUMO energy gap (∆E) as eV for the studied structures.

Structural	TDM (Debye)	∆E (eV)	LUMO (eV)	HOMO (eV)
PVA	7.147	7.334	1.078	−6.255
ZnO	5.550	2.377	−4.252	−6.629
GO	2.373	3.373	−2.524	−5.897
PVA/ZnO	15.733	3.550	0.121	−3.429
PVA/OZn	10.196	1.302	−4.909	−6.211
PVA/ZnO/GO	9.038	2.260	−3.887	−6.147
PVA/OZn/GO	12.243	1.075	−3.873	−4.948

## Molecular electrostatic potential (MESP) maps

**Fig. 5:**
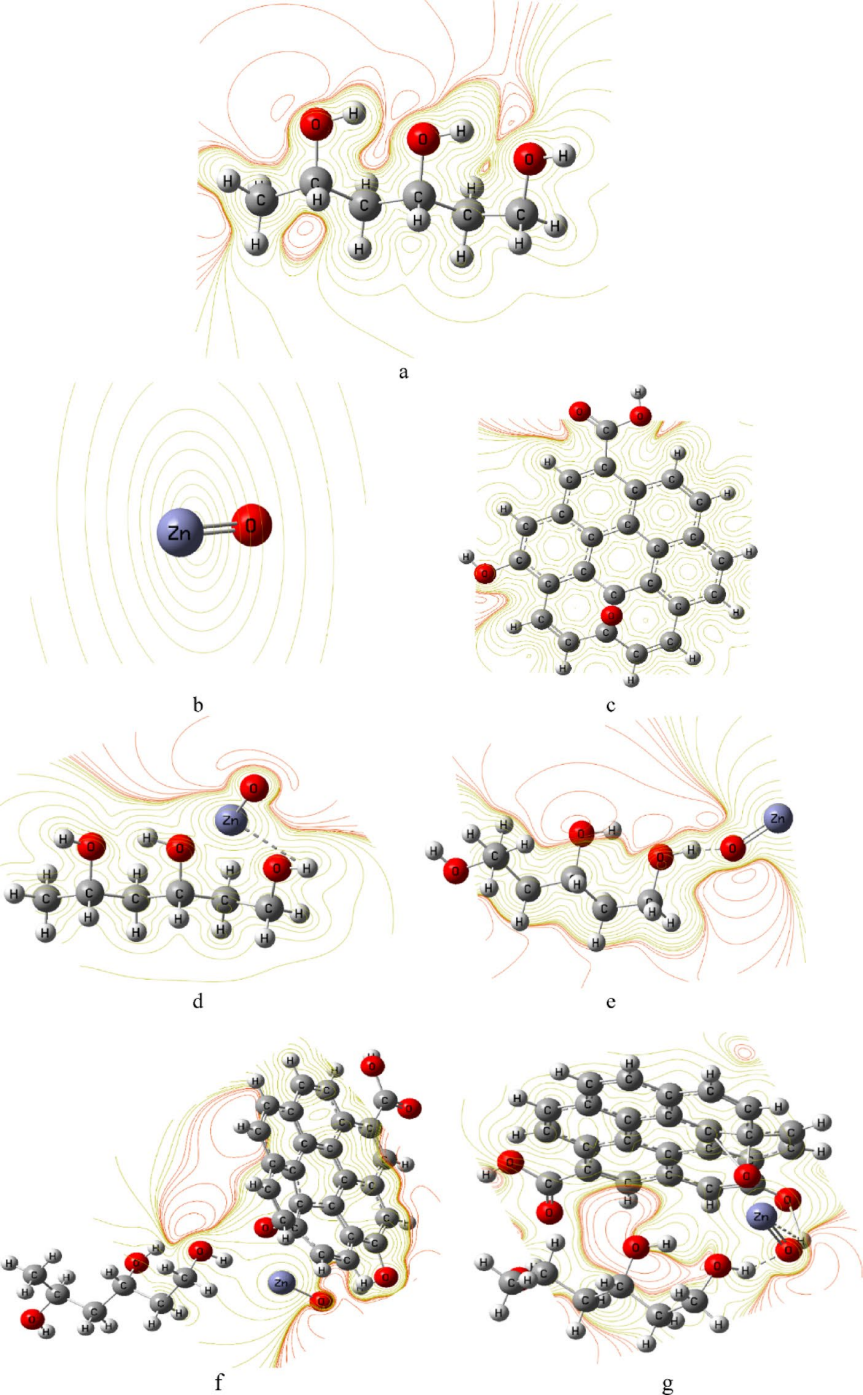
B3LYP/LanL2DZ calculated MESP for (**a**) PVA, (**b**) ZnO, (**c**) Graphene oxide (GO), (**d**) PVA/ZnO, (**e**) PVA/OZn, (**f**) PVA/ZnO/GO and (**g**) PVA/OZn/GO.

The charge distribution inside molecules and materials can be better understood thanks to MESP maps. The color gradient used in the MESP maps follows a scientific standard, going from red (nucleophilic sites, regions of high electron density) to yellow/green (moderate potential) and finally blue (electrophilic sites, regions of low electron density). comprehension reactivity, stability, and molecular interactions require comprehension of these maps. In Fig. 5, the MESP computations for PVA, ZnO, and GO were shown, along with their composites that were created by interactions between either zinc or oxygen atoms.

According to the findings, the O-atom of ZnO and the hydroxyl (OH) groups of the composites have the most advantageous distribution charge. The PVA/OZn/GO (Fig. 5-g) system exhibits the best MESP distribution among the examined composites, suggesting improved stability and stronger intermolecular interactions. These discoveries are further supported by examination of HOMO-LUMO energies, which shows that superior electronic characteristics are reflected in the enhanced charge distribution, which correlates with an adequate energy gap. These results corroborate the link between charge distribution and electrical behavior in the composites investigated and are in good agreement with the data shown in Table 1.

### Density of states calculations

**Fig. 6:**
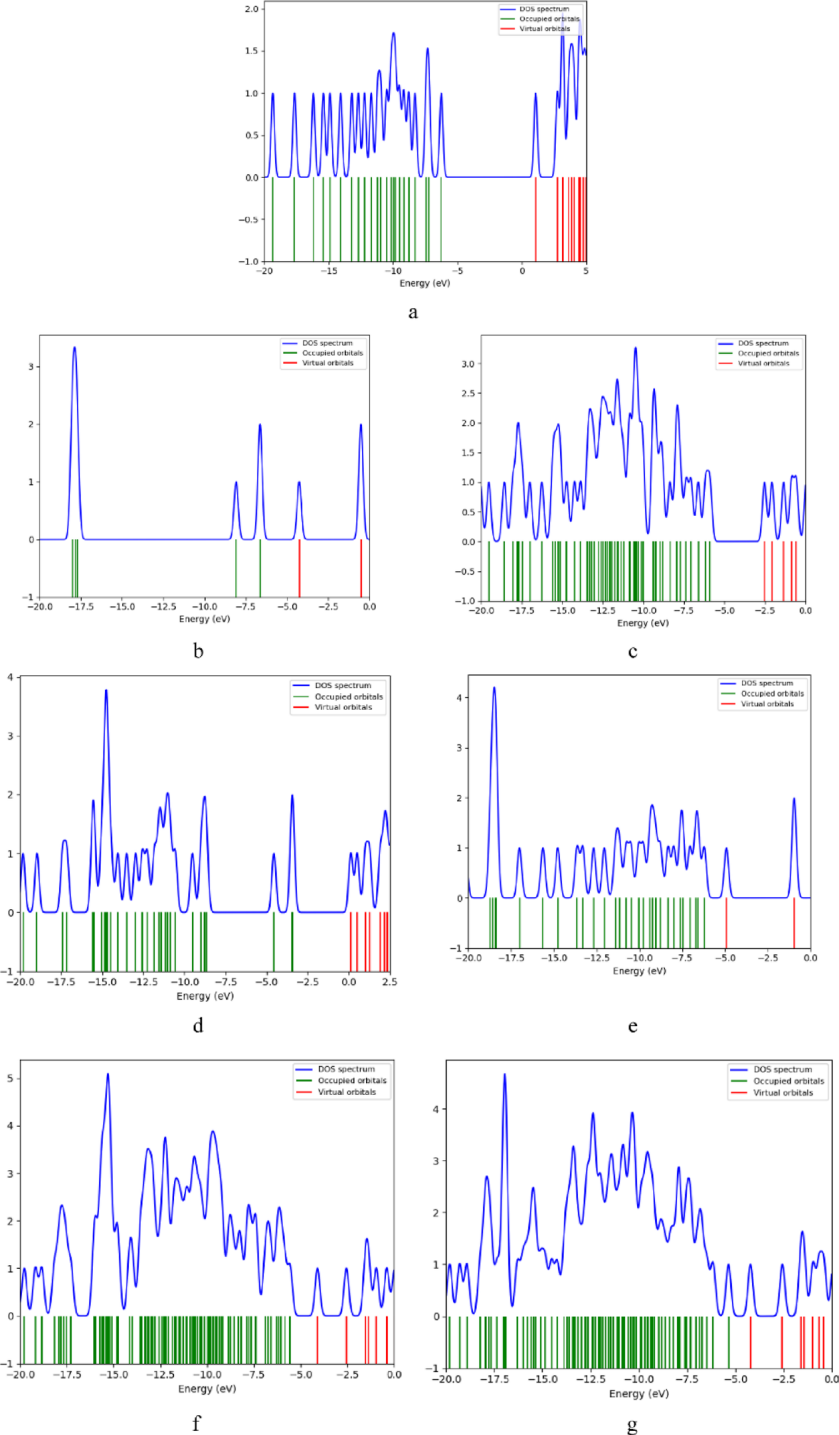
B3LYP/LanL2DZ calculated DOS for (**a**) PVA, (**b**) ZnO, (**c**) GO, (**d**) PVA/ZnO, (**e**) PVA/OZn, (**f**) PVA/ZnO/GO and (**g**) PVA/OZn/GO.

The number of accessible electronic states at each energy level is shown by the DOS. The occupied molecular orbitals in the figure are shown by green lines, and the vacant (virtual) molecular orbitals are shown by red lines. The DOS plot provides information about the electronic characteristics and chemical reactivity of the structures by displaying the energy difference between the lowest unoccupied molecular orbital (LUMO) and the highest occupied molecular orbital (HOMO). Better electrical conductivity and more chemical reactivity are often indicated by a smaller HOMO-LUMO, while greater stability and less reactivity are suggested by a larger energy gap. The DOS for the examined structures, which was calculated using DFT at the B3LYP/LanL2DZ level, is displayed in Fig. 6. The DOS analysis of pure PVA, as shown in Fig. 6-a, is consistent with the theoretical calculations given in Table 4 and displays distinct full and empty energy levels with a wide energy gap. This large energy gap suggests that pure PVA exhibits good insulation properties. Figures 6-d and -e demonstrate the discernible decrease in the energy gap that occurs when ZnO nanoparticles are added to the PVA matrix. This is explained by the contact between the polymer chains and the ZnO surface states, which results in the creation of localized energy levels within the energy gap. This interaction lowers the energy needed for electronic transitions and promotes charge carrier movement. The DOS for ZnO and G are shown in Figs. 6-b and -c, respectively.

As seen in Figs. 6-f and -g, the electrical structures of the PVA/ZnO composite are further modified by the addition of graphene oxide (GO). With its rich π-conjugated system and functional groups that contain oxygen, GO improves interfacial contact and functions as an effective charge transfer medium. This promotes better charge delocalization and significantly narrows the energy gap by improving overlap between the electronic states of PVA and ZnO.

The most notable decrease in the energy gap is seen when ZnO is connected to the polymer via the oxygen atom (Fig. 6-g), indicating that ZnO orientation and bonding configuration are important factors in adjusting the electrical structure of the composite. The observed energy gap tuning is probably a result of enhanced orbital hybridization and charge transfer at the interface made possible by this arrangement.

## Quantum theory of atoms in molecules (QTAIM) topology

**Fig. 7:**
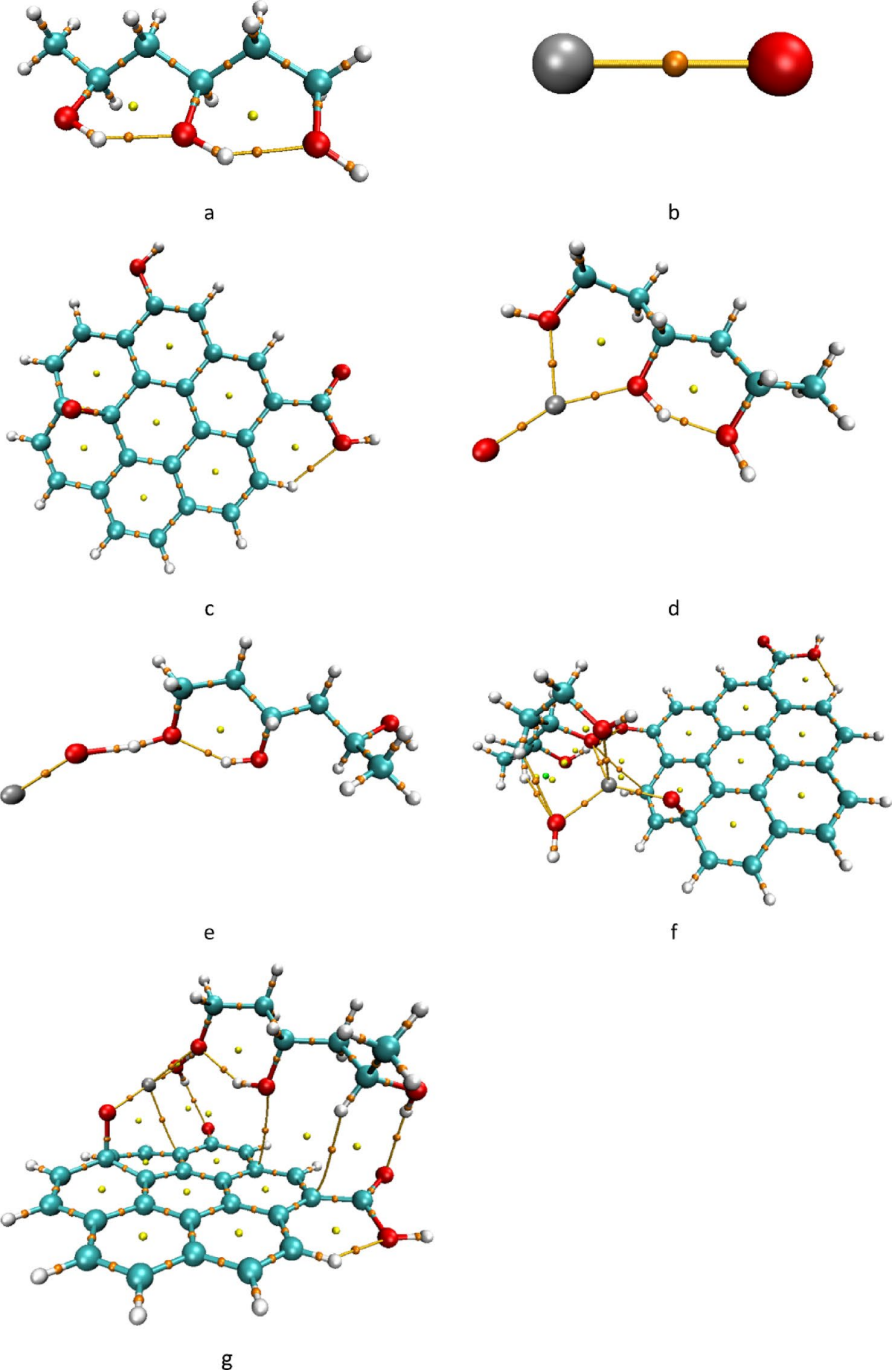
QTAIM topology for studied structures whereas: (**a**) PVA, (**b**) ZnO, (**c**) GO, (**d**) PVA/ZnO, (**e**) PVA/OZn, (**f**) PVA/ZnO/GO and (**g**) PVA/OZn/GO.

QTAIM is a well-known technique that provides thorough understanding of the electronic density distribution by locating crucial sites and bond routes related to both bonds and orders. The nature of the interactions within the PVA–GO–ZnO system was further elucidated using QTAIM analysis, as shown in Table 2; Fig. 7. Electron densities at the bond critical points (ρ(r)) and their Laplacians (∇²ρ(r)), along with the total energy densities (H(r)), provide a quantitative basis for classifying these interactions. The O–H bonds in PVA exhibit high ρ(r) and negative ∇²ρ(r) values, consistent with a covalent character, while the Zn–O bonds show moderate ρ(r) and slightly positive ∇²ρ(r), indicative of partial covalency. In contrast, H···O hydrogen bonds between PVA and GO and C···O contacts exhibit lower ρ(r), positive ∇²ρ(r), and small or slightly positive H(r), reflecting weak, noncovalent interactions. These observations confirm the coexistence of covalent, partially covalent, and weak van der Waals interactions, providing a rigorous, quantitative foundation for the previously described “covalent-like” and hydrogen-bonding interactions in the composite system^43–45^. For the structures under study, Fig. 7 displays the QTAIM topology, which includes bond critical spots and bond routes. In Fig. 7, PVA/ZnO exhibits two hydrogen bonds with the Zn atom, whereas PVA/OZn forms a covalent-like link with the ZnO O atom. Because of the newly established hydrogen bonds, PVA/OZn/GO exhibits more stability than PVA/ZnO/GO.

**Table 2:** QTAIM parameters (ρ, ∇²ρ, H) at bond critical points, with corresponding interaction character, for key PVA–GO–ZnO contacts.

Bond	ρ(*r*) (eV/Å³)	∇²ρ(*r*) (eV/Å⁵)	H(*r*) (eV/Å³)	Interaction Character
O–H (PVA)	5.840	–6.270	–5.440	Covalent
H···O (PVA–GO)	3.260	2.990	0.540	Hydrogen bond/Weak
Zn–O (ZnO–PVA)	4.900	1.360	–0.270	Partially covalent
C···O (GO–PVA)	2.450	2.180	0.140	van der Waals/Weak

## Non-covalent interaction (NCI) and reduced density gradient (RDG)

**Fig. 8:**
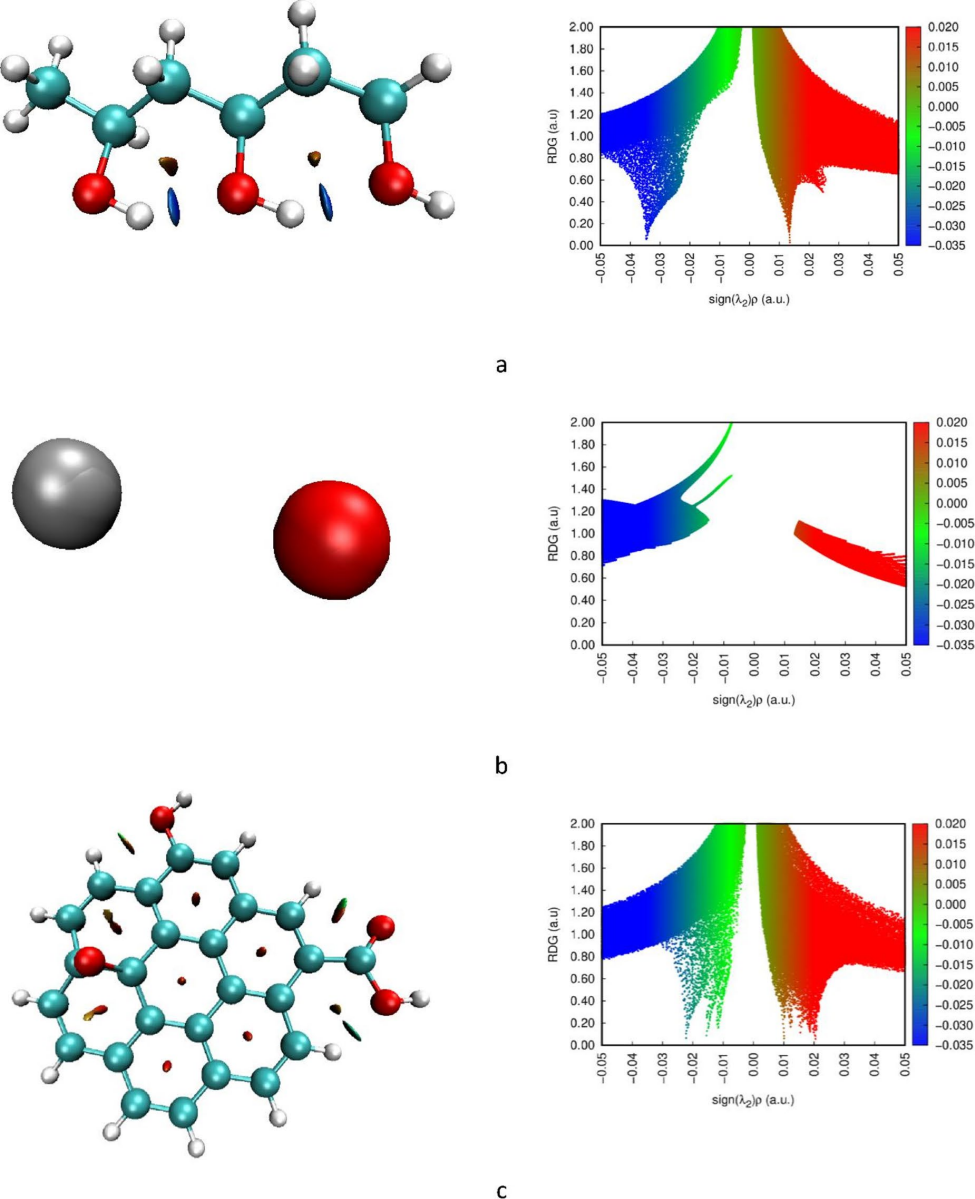

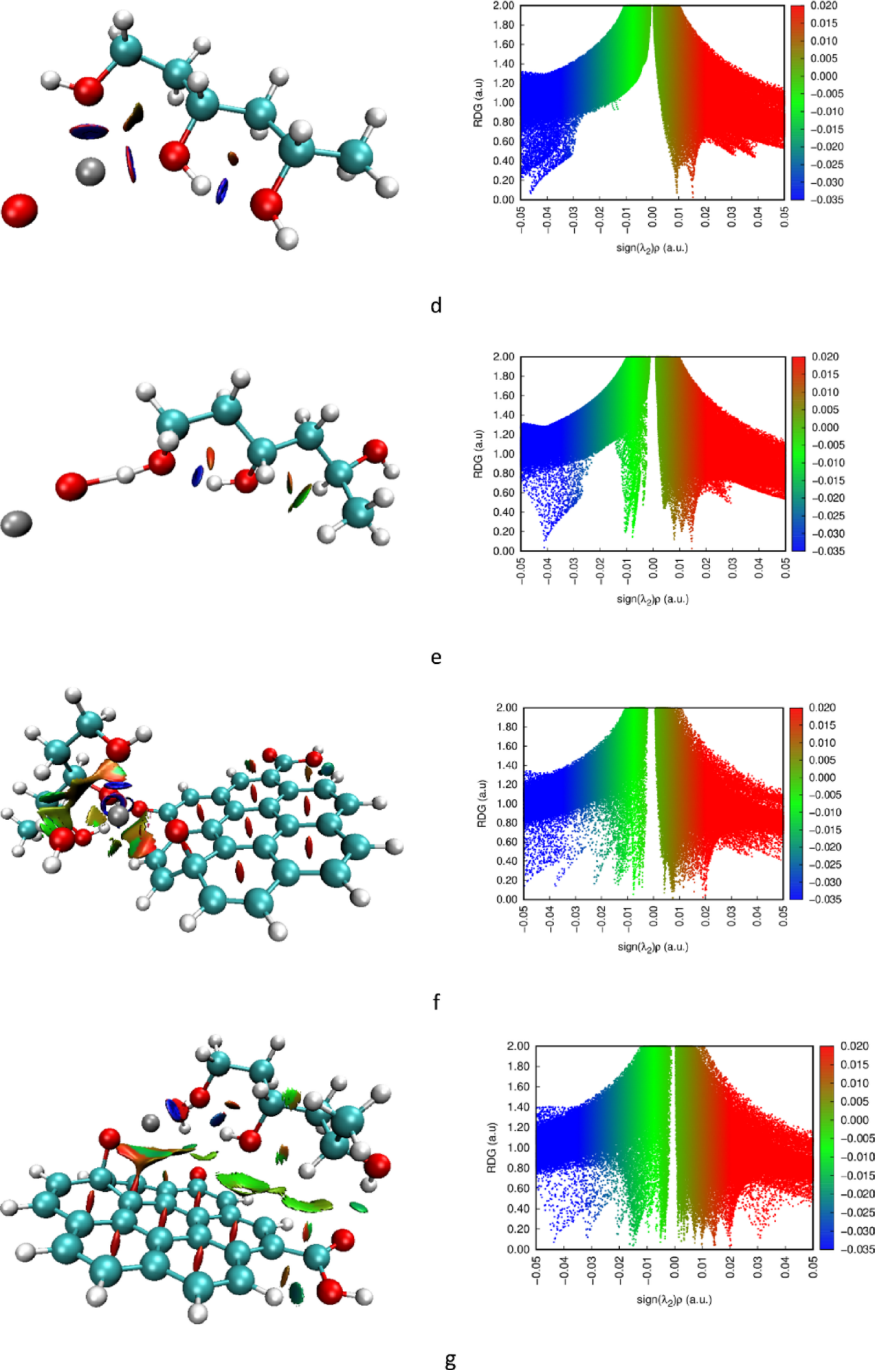
NCI and RDG plots for studied structures whereas: (**a**) PVA, (**b**) ZnO, (**c**) GO, (**d**) PVA/ZnO, (**e**) PVA/OZn, (**f**) PVA/ZnO/GO and (**g**) PVA/OZn/GO.

Reduced Density Gradient (RDG) mapping was carried out in conjunction with Non-Covalent Interaction (NCI) research to better clarify the intermolecular interactions within the composites. These techniques use gradient plots and color-coded isosurfaces to illustrate weak non-bonded interactions including hydrogen bonding, van der Waals forces, and steric repulsions^46^. Strong steric repulsion is indicated by red regions in NCI-RDG plots (Fig. 8), weaker dispersive forces (van der Waals) are indicated by green regions, and strong attractive interactions (hydrogen bonds) are indicated by blue regions.

A pronounced blue signal in the RDG spectrum confirms the hydrogen bonding between the oxygen atom of PVA and a hydrogen atom (blue isosurface) shown in the NCI plot (Fig. 8-a). NCI is quite low for ZnO alone (Fig. 8-b). As a result of π-π stacking distortions, GO displays noticeable red isosurfaces between its aromatic rings (Fig. 8-c), which indicate steric repulsion.

PVA interacts with ZnO through the O and Zn atoms. In Fig. 8-d, the Zn and O atoms of PVA exhibit significant NCI attractions, while in Fig. 8-e, the PVA/OZn example exhibits a weak van der Wall interaction within PVA, as indicated by the green and RDG green spikes. Similar behavior is shown by PVA/ZnO/GO and PVA/OZn/GO (Figs. 8-f and -g). However, PVA/OZn/GO has a higher NCI, a slightly stronger attraction that is countered by a little stronger repulsion, and weaker contacts, which can stabilize it more than the PVA/ZnO/GO instance.

### Calculated reactivity descriptors

Table 3 presents the DFT/B3LYP/LanL2DZ calculated global reactivity descriptors for the investigated structures PVA, ZnO, GO, and their nanocomposites. The Ionization Potential (I) values indicate the ease of electron removal. Among the studied systems, PVA/ZnO exhibits the lowest I = 3.429 eV, implying enhanced electron-donating ability, which can promote charge transfer during sensing processes. Conversely, PVA/OZn shows the highest I = 6.211 eV among the composites, reflecting greater electronic stability. The electron affinity (A), a measure of the tendency to accept electrons, is significantly higher for PVA/OZn (4.909 eV) and PVA/OZn/GO (3.893 eV) compared to pristine PVA (−1.078 eV), suggesting that incorporation of OZn and GO considerably enhances electron-accepting capability, an essential feature for interaction with electron-rich analytes. The electronic chemical potential (µ) values follow a similar trend, with PVA/OZn recording the highest (5.560 eV), indicating strong electron-attracting tendencies and better charge redistribution upon adsorption of gas or humidity molecules. The chemical hardness (η) values reveal that PVA/OZn and PVA/OZn/GO possess the lowest hardness (0.650 eV and 0.537 eV), corresponding to the highest absolute softness (S) values (1.537 eV⁻¹and 1.861 eV⁻¹) respectively. This high softness implies increased chemical reactivity and polarizability, making these systems more responsive to external stimuli. Finally, the electrophilicity index (ω), which quantifies the stabilization energy upon acquiring additional electronic charge, is markedly enhanced in PVA/OZn (23.751 eV) and PVA/OZn/GO (18.103 eV). These elevated ω values confirm that OZn-containing composites are highly electrophilic, and may be a desirable feature for sensing applications where interaction with nucleophilic species is required. So that, the PVA/OZn/GO composite exhibits a synergistic enhancement in electron affinity, chemical softness, and electrophilicity, leading to improved overall chemical reactivity. These modifications are anticipated to markedly enhance the sensing performance in gas and humidity sensing applications, aligning well with previously reported results for similar systems^19,29^.

**Table 3:** DFT/B3LYP/LanL2DZ calculated global reactivity descriptors: Ionization Potential (I), Electronic Affinity (A), Electronic chemical potential (µ), Chemical hardness (η), Absolute softness (S) and Electrophilicity index (ω) for the studied structures.

Structure	I (eV)	A (eV)	µ (eV)	η (eV)	S (eV⁻¹)	ω (eV)
PVA	6.255	−1.078	2.589	3.667	0.273	0.914
ZnO	6.629	4.252	5.440	1.188	0.841	12.454
GO	5.897	2.524	4.210	1.687	0.593	5.256
PVA/ZnO	3.429	−0.121	1.654	1.775	0.563	0.771
PVA/OZn	6.211	4.909	5.560	0.650	1.537	23.751
PVA/ZnO/GO	6.147	3.887	5.017	1.130	0.885	11.136
PVA/OZn/GO	4.948	3.873	4.411	0.537	1.861	18.103

### TDM, energy gap and MESP for the interaction between PVA/ZnO/GO and H_2_O and CO_2_

For a variety of systems, including individual molecules (H_2_O, CO₂) and polymer nanocomposites based on PVA/ZnO/GO and PVA/OZn/GO with varying adsorbates of H2O and CO₂. Table 4 displays the TDM and the energy gap. This is evident from the data. With very low or zero TDM values and high energy gaps (~ 9.8–9.9 eV), H₂O and CO₂ show poor electronic transitions and limited optical activity. Particularly for PVA/OZn/GO-CO_2_ (ΔE = 0.539 eV), PVA/ZnO/GO composites exhibit a notable drop in ΔE as they interacted with CO₂, indicating improved optical qualities and increased electronic conductivity.

Additionally, this structure has a high TDM (14.264 Debye), which suggests robust TDM and improved absorption of light. In comparison to PVA/ZnO/GO, PVA/OZn/GO composites have slightly lower ΔE values and a wider range of TDM values (6.341–14.264 Debye). In comparison to PVA/OZn/GO-CO_2_, the structure PVA/ZnO/GO-2H_2_O exhibits high TDM (10.556 Debye) and a modest ΔE (1.322 eV), indicating robust optical activity but somewhat lower electrical conductivity.

Based on the theoretical calculations, PVA/OZn/GO–CO₂ is the most promising material for optoelectronic performance, as it combines a high TDM (for strong optical transitions) with a low ΔE (for enhanced electronic properties). Moreover, it may also be considered suitable for sensing applications due to its strong optical activity. However, PVA/ZnO/GO–2 H₂O is particularly better for sensing, especially humidity sensors, because it exhibits a high TDM.

**Table 4:** B3LYP/LanL2DZ calculated TDM as Debye; HOMO/LUMO energy gap (∆E) as eV for the PVA/ZnO/GO and PVA/OZn/GO Nanocomposites interacted with of H₂O, CO₂.

Structural	TDM (Debye)	ΔE (eV)
H_2_O	2.462	9.811
CO_2_	0.000	9.920
PVA/ZnO/GO-H_2_O	7.062	0.668
PVA/ZnO/GO-2H_2_O	10.556	1.322
PVA/ZnO/GO-CO2	8.5059	0.602
PVA/ZnO/GO-2CO_2_	9.635	0.647
PVA/OZn/GO-H_2_O	9.761	0.757
PVA/OZn/GO-2H_2_O	13.895	1.542
PVA/OZn/GO-CO_2_	14.264	0.539
PVA/OZn/GO-2CO_2_	6.341	0.800

**Fig. 9:**
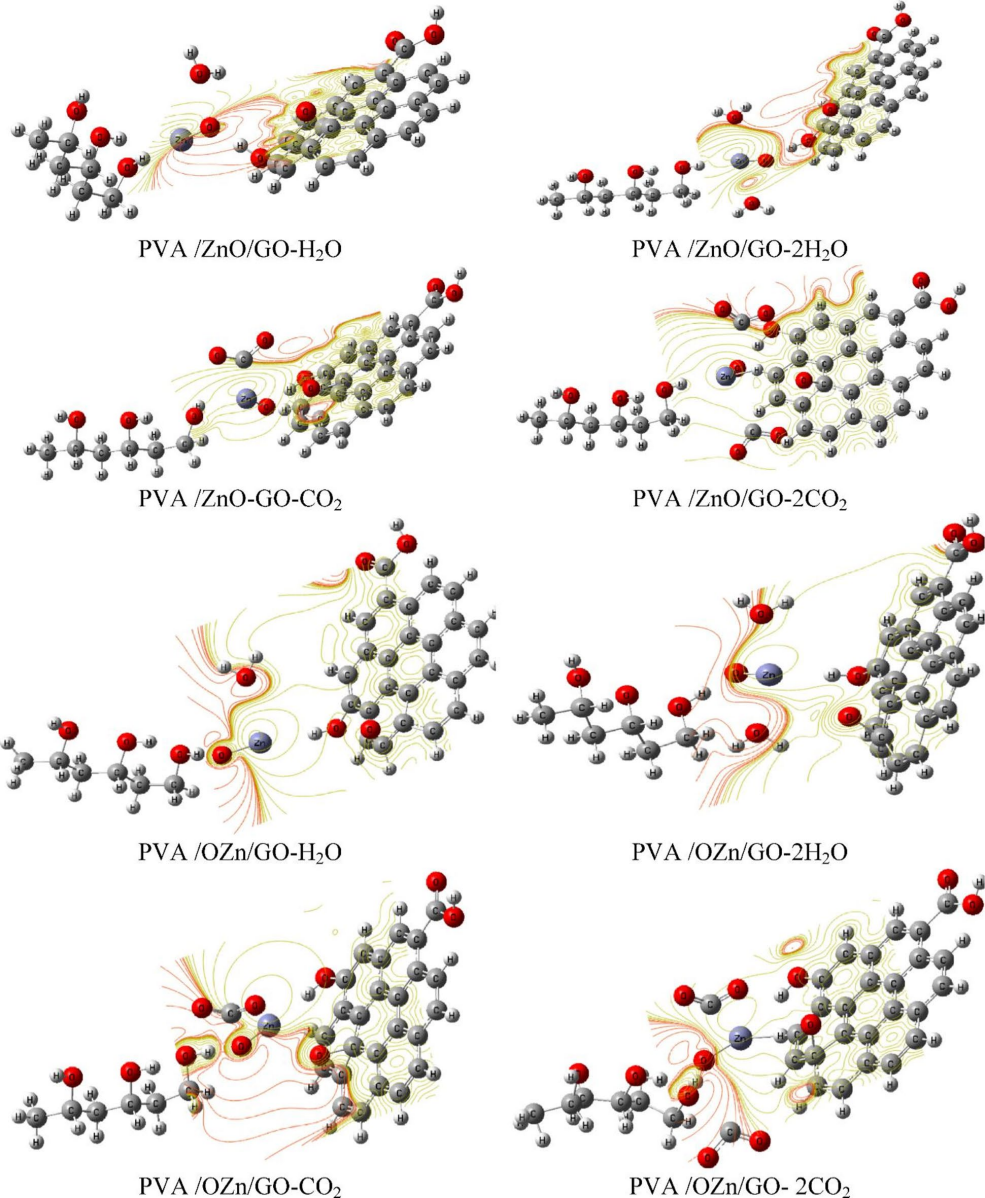
B3LYP/LanL2DZ calculated MESP for PVA/ZnO-GO-CO_2_ PVA/OZn/GO nteracted with H_2_O, 2 H_2_O, CO_2_ and 2CO_2_ as humidity and gas sensing.

Figure 9 shows the calculated molecular electrostatic potential (MESP) maps at the B3LYP/LanL2DZ level for PVA/ZnO/GO and PVA/OZn/GO nanocomposites interacting with H₂O, 2 H₂O, CO₂, and 2CO₂ molecules. In these maps, red regions represent electron-rich (negative potential) areas, blue regions indicate electron-deficient (positive potential) areas, and yellow/orange contours correspond to intermediate potentials. The color distributions confirm that all composites undergo notable charge redistribution upon adsorption of H₂O and CO₂, indicating their overall sensitivity to humidity and gases. Among all cases, the PVA/OZn/GO composite interacting with H_2_O and CO_2_ molecules shows the most intense red contrast and the broadest high-potential regions, indicating the strongest polarization and activation. This is consistent with its highest total dipole moment and its relatively small energy gap for PVA/OZn/GO-H_2_O and PVA/OZn/GO-CO_2_ respectively, which together suggest enhanced charge transfer and reactivity. These features indicate that the PVA/OZn/GO composite is sensitive to both gases and humidity, with markedly higher reactivity and sensitivity when exposed to CO_2_ molecules.

### Adsorption Energy for the interaction between PVA/ZnO/GO and H_2_O and CO_2_

One important metric for assessing how firmly an atom or molecule adheres to the surface is adsorption energy. The effectiveness of PVA/ZnO/GO as a humidity and gas sensor was further assessed by computing the adsorption energies for the optimized structures. The significance of adsorption energy is determined by analyzing the substance’s surface potential, sensitivity, and selectivity to adsorb any other material. Thus, estimating the adsorption energy is crucial for evaluating materials and determining which ones promising for sensing applications^47^. The adsorption energy is the difference between the final system energy of the two materials and their separate energy sources before contact. The adsorption energy was obtained using the following Eqs^29,48^. :

E_Ads_= [E_System_ - (E_Adsorbent +_ E_Adsorbate_)]

To assess the applicability of PVA/ZnO/GO and PVA/OZn/GO nanocomposites as a humidity and gas sensor. Table 5 displays the calculated adsorption energies for these nanocomposites in the presence of water and CO_2_ molecules. All examined structures show negative total energy, as shown in the table, demonstrating their thermodynamic structural stability. The adsorption energy values for carbon dioxide (CO_2_) and water (H_2_O) molecules are notably always negative, ranging from − 0.188 eV to −0.406 eV, indicating that the adsorption processes are exothermic and energetically beneficial. The PVA/OZn/GO configuration has a much better affinity for gas molecules than the typical PVA/ZnO/GO structure, according to a comparison analysis. In particular, CO_2_ molecules adsorption on the PVA/OZn/GO surface produced the maximum adsorption energy of −0.406 eV, indicating a stronger binding strength that may be related to improved charge redistribution at the O-terminated interface. Notably, the PVA/ZnO/GO structure also displays significant and steady adsorption energy values (e.g., −0.229 eV for CO_2_), even though the PVA/OZn/GO configuration exhibits the maximum binding strength. With a balanced interaction that enables both target capture and effective surface recovery, these data show that PVA/ZnO/GO is an extremely sensitive and promising choice for gas detection.

Additionally, the data show that CO_2_ is clearly preferred over H_2_O; for example, the E_Ads_ for CO_2_ on the PVA/OZn/GO composite are around 32% greater than those for H_2_O in the single-molecule phase. The materials promise as a high-performance gas sensor is crucially shown by its improved sensitivity and selectivity towards CO_2_ and H_2_O. The energy levels are within the physisorption domain, suggesting that although the contact is enough for measurable electronic changes; it is still reversible, which is crucial for the sensing platform’s cyclic recovery and reusability.

Although these results offer a solid theoretical foundation for comprehending molecular charge transfer and binding mechanisms, experimental confirmation is still crucial.

**Table 5:** Calculated total energy and adsorption energy for the studied structures of PVA/ZnO/GO and interacted with H_2_O and CO_2_.

Structure	Total energy (eV)	Adsorption energy (eV)
H_2_O	−2079.332	
2H_2_O	−4158.664	
CO_2_	−5130.437	
2CO_2_	−10260.875	
PVA/ZnO/GO	−50718.703	
PVA/OZn/GO	−50726.703	
PVA/ZnO/GO-H_2_O	−52798.245	−0.210
PVA/ZnO/GO-2H_2_O	−54877.555	−0.188
PVA/ZnO/GO-CO_2_	−55849.369	−0.229
PVA/ZnO/GO-2CO_2_	−60979.810	−0.232
PVA/OZn/GO-H_2_O	−52806.341	−0.306
PVA/OZn/GO-2H_2_O	−54885.608	−0.241
PVA/OZn/GO-CO_2_	−55857.521	−0.381
PVA/OZn/GO-2CO_2_	−60987.984	−0.406

To obtain quantitative evidence of the electronic interaction of CO₂/H₂O with PVA/ZnO/GO nanocomposite material, the charge rearrangement that takes place according to our previously acquired MESP and QTAIM results has been evaluated.

Although NCI analysis and QTAIM analysis give qualitative information about the bond formation process, quantitative evidence is provided by the drastic change in the electronic reactivity parameters. The decrease in the chemical potential value and an increase in global electrophilicity quantify the ability of the surface to accept/donate electrons.

The interaction energy value of −0.381 eV for the CO2 adsorbed complex, along with the specific pattern of electron-density distribution at the bond critical points (QTAIM), signifies that the stable state of physisorption exists. Such values of interaction energies can be typical for the sensors which are highly sensitive to changes and possess fast and reversible recovery abilities because this value of energy is enough to induce the bandgap transition from 1.075 eV to 0.539 eV. The results obtained from the changes of MESP surface potential signify that there is a noticeable charge transfer from the ZnO/GO active sites to the CO2 molecule, which can be considered as a chemo-resistive response mechanism.”

The incorporation of ZnO and GO into PVA creates a so called synergistic multifunctional nanocomposite. The GO provides the “conductive networks” and ZnO provides the “surface active sites” when combined together reduced optical band gap and favorable charge redistribution” observed. This interpretation aligns with recent DFT study on PVA/CuO/GO hybrid nanostructures^28^, which demonstrated that moderate adsorption energies offer an optimal balance between adsorption strength and desorption efficiency. This kind of behavior is especially wanted for chemiresistive gas sensors, since it guarantees enough interaction with gas molecules while allowing for quick recovery and high reversibility. The reduced optical band gap, DOS redistribution, and the fast response–recovery characteristics observed experimentally support the same sensing mechanism in the current PVA/ZnO/GO system.

Moreover, the significant electronic modulation throughout the drop in HOMO-LUMO gap) induced only by CO₂ adsorption serves as a distinct sensing signal that allows the sensor to differentiate CO₂ from H₂O.

## Conclusion

This research offers an in-depth examination via density functional theory (DFT) of the structural, electronic, and adsorption characteristics of PVA/ZnO/GO nanocomposites, as well as their possible use in gas and humidity sensing. The generated FTIR spectra, along with MESP, QTAIM, and NCI-RDG analyses, validated the establishment of robust interfacial connections such as hydrogen bonds and van der Waals forces that aid in structural stabilization and promote charge transfer throughout the composite network.

The electronic properties of PVA were significantly altered by the addition of ZnO and GO, resulting in a considerable decrease in the HOMO-LUMO energy gap from 7.334 eV for pure PVA to 1.075 eV for the PVA/OZn/GO nanocomposite, as well as an increase in the total dipole moment from 7.147 Debye to 12.243 Debye. The modifications point to greater electronic polarization, better charge delocalization, and enhanced surface reactivity all of which are favorable characteristics for sensing materials.

The adsorption calculations indicated that interactions with both H_2_O and CO_2_ molecules are thermodynamically favorable, producing adsorption energies of −0.306 eV and − 0.381 eV, respectively. The PVA/OZn/GO-CO_2_ complex demonstrated the most robust electronic response among the analyzed configurations, as evidenced by its minimal energy gap (0.539 eV) and maximal dipole moment (14.264 Debye), signifying a marked charge redistribution upon adsorption. On the other hand, the PVA/ZnO/GO-2H_2_O system exhibited a relatively high dipole moment, emphasizing its potential sensitivity to humidity detection.

The results obtained demonstrate a clear connection between interfacial interactions, modulation of electronic structure, and adsorption behavior in PVA/ZnO/GO nanocomposites. The results show that the interaction route between the polymer matrix and ZnO, via either the Zn or O atom, is essential for controlling charge-transfer processes and sensing-related characteristics. Overall, the molecular-level insights obtained in this study, together with the synergistic incorporation of ZnO and GO into the PVA matrix provide a promising strategy for tailoring the structural and electronic properties of the nanocomposites, enabling the development of high-performance, low-cost, and sustainable materials for next-generation CO₂ gas and humidity sensing applications.

In addition, the obtained values of the reactivity parameters and the change in the HOMO-LUMO gap clearly demonstrate that the underlying sensing mechanism occurs due to the charge transfer process at the PVA/ZnO/GO interface caused by the exposure to gases. These results ensure the reliable theoretical basis and demonstrate the high potential of the proposed nanocomposite for CO2 detection.

## Data Availability

The data supporting the findings of this study can be obtained from the corresponding author upon request, subject to reasonable conditions.
